# Quantitative and Compositional MRI of the Articular Cartilage: A Narrative Review

**DOI:** 10.3390/tomography10070072

**Published:** 2024-06-24

**Authors:** Domenico Albano, Umberto Viglino, Francesco Esposito, Aldo Rizzo, Carmelo Messina, Salvatore Gitto, Stefano Fusco, Francesca Serpi, Benedikt Kamp, Anja Müller-Lutz, Riccardo D’Ambrosi, Luca Maria Sconfienza, Philipp Sewerin

**Affiliations:** 1 IRCCS Istituto Ortopedico Galeazzi, 20161 Milan, Italy; carmelomessina.md@gmail.com (C.M.); sal.gitto@gmail.com (S.G.); stefano.fusco@unimi.it (S.F.); fr.serpi@gmail.com (F.S.); riccardo.dambrosi@hotmail.it (R.D.); io@lucasconfienza.it (L.M.S.); 2 Dipartimento di Scienze Biomediche, Chirurgiche ed Odontoiatriche, Università degli Studi di Milano, 20122 Milan, Italy; 3 Unit of Radiology, Ospedale Evangelico Internazionale, 16100 Genova, Italy; umberto.viglino@gmail.com; 4 Division of Radiology, Department of Precision Medicine, Università degli Studi della Campania Luigi Vanvitelli, 80138 Naples, Italy; fraespo2312@gmail.com; 5 Postgraduate School of Diagnostic and Interventional Radiology, Università degli Studi di Milano, Via Festa del Perdono 7, 20122 Milan, Italy; rizzo.aldo.95@gmail.com; 6 Dipartimento di Scienze Biomediche per la Salute, Università degli Studi di Milano, 20122 Milan, Italy; 7 Department of Diagnostic and Interventional Radiology, Medical Faculty and University Hospital Düsseldorf, Heinrich Heine University Düsseldorf, 40225 Düsseldorf, Germany; benedikt.kamp@med.uni-duesseldorf.de (B.K.); anja.lutz@med.uni-duesseldorf.de (A.M.-L.); 8 Rheumazentrum Ruhrgebiet, Ruhr University Bochum, 44649 Herne, Germany; philipp.sewerin@med.uni-duesseldorf.de; 9 Department and Hiller-Research-Unit for Rheumatology, Medical Faculty, Heinrich-Heine-University Düsseldorf, 40225 Düsseldorf, Germany

**Keywords:** magnetic resonance imaging, cartilage, quantitative, mapping, osteoarthritis

## Abstract

This review examines the latest advancements in compositional and quantitative cartilage MRI techniques, addressing both their potential and challenges. The integration of these advancements promises to improve disease detection, treatment monitoring, and overall patient care. We want to highlight the pivotal task of translating these techniques into widespread clinical use, the transition of cartilage MRI from technical validation to clinical application, emphasizing its critical role in identifying early signs of degenerative and inflammatory joint diseases. Recognizing these changes early may enable informed treatment decisions, thereby facilitating personalized medicine approaches. The evolving landscape of cartilage MRI underscores its increasing importance in clinical practice, offering valuable insights for patient management and therapeutic interventions. This review aims to discuss the old evidence and new insights about the evaluation of articular cartilage through MRI, with an update on the most recent literature published on novel quantitative sequences.

## 1. Introduction

Cartilage tissue can be categorized histologically based on molecular composition into hyaline, elastic, and fibrocartilaginous cartilage. Hyaline cartilage is the most prevalent type. It covers the cortical bone within the joints and can be subdivided into four zones: the superficial zone, transitional zone, radial zone, and calcified cartilage zone [1]. Injuries or degeneration may determine the degradation of articular cartilage, which can subsequently lead to severe joint pain, reduced functionality, and the development of osteoarthritis (OA). Thanks to its contrast resolution in soft tissue, magnetic resonance imaging (MRI) is the best imaging method for evaluating articular cartilage. As a matter of fact, MRI is essential for the diagnosis of chondral injuries, but also for assessing post-treatment changes after surgery or conservative therapies. Rapid technological advancement have led to the introduction of novel MRI techniques, sequences, and applications. Awareness of these novelties is essential, as it will allow us to walk hand in hand with new tools that can be employed in research and clinical practice. This article arises from the need for continuous updates on this topic with the aim of discussing the old evidence and new insights about the evaluation of articular cartilage through MRI, thereby providing a comprehensive review on the most recent literature published on novel quantitative sequences. To achieve this, we have not performed a systematic review, but instead conducted a narrative review checking several articles written in English on the topic “MRI of the cartilage” retrieved from different databases (PubMed, Web of Science, and SCOPUS) without limitations in terms of the year of publication.

## 2. MRI of the Articular Cartilage

### 2.1. Conventional MRI

**Figure 1:**
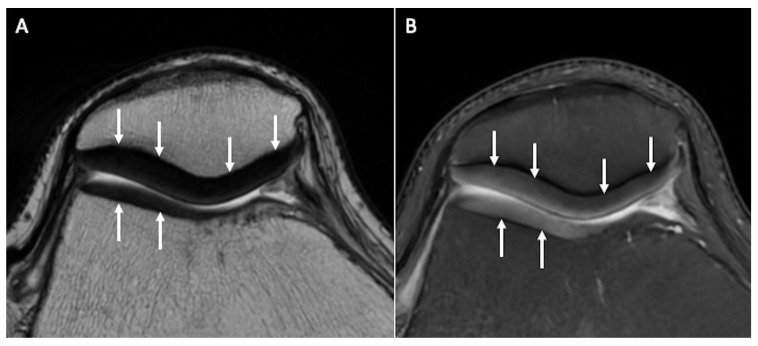
Normal signal and thickness of patellar and trochlear cartilage (arrows) on axial T2w (**A**) and axial fat-saturated PDw (**B**) images.

To date, musculoskeletal MRI frequently relies on two-dimensional (2D) multislice acquisitions obtained in various planes. These acquisitions are typically conducted using turbo or fast spin-echo (FSE) techniques, which offer impressive signal-to-noise ratios and tissue contrast. In a recent meta-analysis, it was found that multiplanar 2D MRI sequences can detect cartilage lesions with combined sensitivity and specificity of 76% and 93%, respectively [2]. However, the inherent anisotropy of the voxels in these 2D acquisitions necessitates the acquisition of data in multiple planes to reduce partial volume artifacts. Proton-density weighted (PDw), intermediate-weighted (Iw), and T2-weighted (T2w) FSE imaging sequences, with or without fat suppression, have been recommended for evaluating the integrity of articular cartilage (Figure 1) [3].

**Figure 2:**
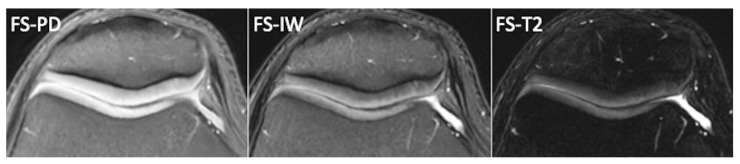
Normal femoro-patellar cartilage of a healthy 29-year-old subject imaged with axial fat-suppressed PDw, fat-suppressed Iw, and fat-suppressed T2w images.

Among them, T2-weighted images provide excellent contrast between joint fluid and the cartilage surface, making them useful for detecting surface defects and cartilage delamination. However, T2-weighted images offer poor contrast between articular cartilage and subchondral bone, making it difficult to detect cartilage thinning and determine the exact depth of cartilaginous lesions. PD-weighted images, on the other hand, offer excellent contrast between joint fluid, articular cartilage, and the underlying bone plate. They are effective in depicting both surface irregularities and deeper cartilage abnormalities, and thus, PD-weighted sequences are commonly used in cartilage imaging. Iw sequences are similar to PDw images but have longer echo times, resulting in more T2-weighting. Some radiologists prefer intermediate-weighted images over PD sequences to improve sensitivity for detecting early cartilage degeneration [4]. Iw sequences offer a favorable balance between the increased sensitivity of PDw sequences to water signal intensity and the heightened specificity of T2w sequences being less susceptible to the magic angle effect than PDw [4]. The varying orientations of collagen fibers create multiple alignments for the internuclear vector of dipolar-coupled water molecules on collagen with the static magnetic field, producing a small dipolar magnetic field at an angle to the external magnetic field. This causes T2 anisotropy, with subsequent variations in signal intensities across different regions of cartilage in T2w images. Consequently, cartilage often exhibits a laminar appearance due to T2 anisotropy. The angular dependence of the dipolar interaction minimizes effects in regions where the fiber orientation is approximately 55° relative to the static magnetic field. This “magic angle effect” results in the disappearance of the laminar appearance in MR images of articular cartilage. Furthermore, FSE FS-Iw sequences should be regarded as the optimal choice for a comprehensive assessment of the entire joint in OA, including bone marrow edema, synovitis/effusion, ligaments, and menisci. Fat-suppression techniques are often employed in cartilage imaging because they enhance sensitivity for subchondral bone marrow lesions associated with cartilage damage and reduce chemical shift artifacts at the cartilage–subchondral bone interface (Figure 2).

Three-dimensional (3D) T1-weighted spoiled gradient-recalled echo (SPGR) acquisitions offer finely detailed, high-resolution images with thin consecutive slices. When combined with fat suppression, they have been recommended to enhance the dynamic range of signal intensity across the entire image, potentially improving the detection of changes in articular cartilage. Xin et al. have demonstrated that the average Whole-Organ Magnetic Resonance Imaging Score (WORMS) score for evaluating cartilage lesions using the SPGR sequence was 3.85 ± 4.72, which was significantly higher than that obtained with T2w images (2.38 ± 3.86, *p* < 0.05) [5].

There are two primary drawbacks associated with this method: the absence of consistent contrast between cartilage and fluid that delineates surface defects (small focal lesions may be obscured), and the extended duration of this sequence, which typically lasts around 8 min [6]. Three-dimensional SPGR imaging is regarded as the standard approach for an evaluation focused on knee cartilage, primarily due to its heightened sensitivity compared to two-dimensional methods; it excels in revealing cartilaginous defects, delivering results similar to those obtained through arthroscopy [7], and has a relevant role in morphometry assessment.

Furthermore, 3D gradient-echo (GRE) techniques were found to be the most effective at identifying and categorizing lesions in hyaline cartilage and subchondral bone associated with rheumatoid arthritis. These techniques exhibited a more robust correlation with macroscopic data in comparison to the SE and FSE sequences (*p* = 0.05) [8]. Newer options for evaluating articular cartilage, such as 3D Turbo spin-echo (TSE) sequences, have become accessible in recent times. The primary benefit of using these sequences instead of 3D GRE pulse sequences lies in its capacity to produce genuine T2 contrast. Advanced acceleration techniques have significantly reduced the time required for data acquisition. These techniques include bidirectional parallel imaging using a shifted CAIPIRINHA pattern and undersampling based on compressed sensing [9]. Modern 3D FSE/TSE pulse sequences have demonstrated enhanced sensitivity and specificity in the detection and characterization of articular cartilage when compared to 3D-GRE techniques [10]. This improvement can be attributed to their advantages, which include I-w tissue contrast [2,10].

Driven equilibrium Fourier transform (DEFT) imaging employs a 90° pulse to realign magnetization with the z-axis, amplifying the signal from tissues with extended T1 relaxation times, such as synovial fluid. In contrast to conventional T1- or T2-weighted MRI, the contrast in DEFT imaging hinges on the T1/T2 ratio of a specific tissue. In the context of musculoskeletal imaging, the DEFT sequence boosts the signal from synovial fluid rather than diminishing the signal from cartilage, as seen in T2-w sequences. Consequently, this leads to a bright appearance of synovial fluid at short TRs (repetition time). When using short TRs, DEFT exhibits more pronounced contrast between cartilage and fluid compared to SPGR, PDw, or T2-w FSE sequences [11].

The Dual Echo Steady State (DESS) is a 3D coherent GRE sequence that simultaneously acquires two or more gradient echoes. Each set of these echoes is separated by a refocusing pulse, and when the data are combined, a stronger T2* weighting is generated, leading to elevated signal intensity in both cartilage and synovial fluid. While there are currently no available studies regarding its application in small joints of rheumatoid arthritis patients, the potential utility of the 3D DESS sequence will likely be of significant interest in future endeavors aimed at imaging small joints affected by rheumatoid arthritis [12]. As we will see later in this article, the DESS sequence has also the potential advantage of providing quantitative information in addition to purely morphological data.

Balanced steady-state free precession (bSSFP) MRI is an effective technique for acquiring high-signal 3D MR images, although it has not been proven to be superior to standard 2D and 3D GRE sequences in the evaluation of knee articular cartilage. Further, it requires a longer acquisition time because multiple acquisitions are needed to produce images with adequate resolution. Depending on the manufacturer of the MRI scanner, this approach may also be referred to the true fast imaging with steady-state precession (trueFISP, Siemens Healthineers, Erlangen, Germany), fast imaging employing steady-state acquisition (FIESTA, GE Healthcare, Chicago, IL, USA), or balanced fast-field echo imaging (Philips Healthcare, Hamburg, Germany) [13]. Last, Vastly Interpolated Projection Reconstruction Imaging (VIPR)-SSFP is an advanced method that combines bSSFP imaging with 3D radial k-space acquisition, allowing the acquisition of images featuring isotropic spatial resolution and T2/T1-w contrast [14].

### 2.2. Cartilage Morphometry

**Figure 3:**
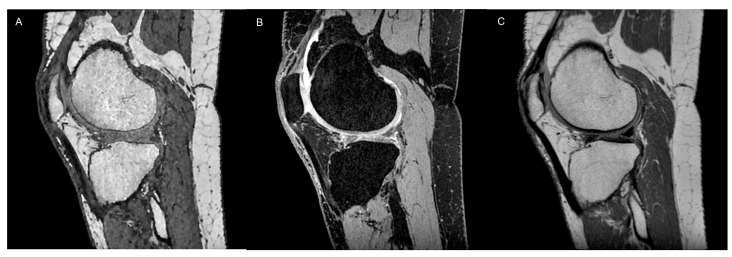
Cartilage morphometry of a 51-year-old male through 3D sequences, which allow quantitative assessment of cartilage morphological characteristics, like a T1-weighted spoiled gradient echo (SPGR, **A**), a 3D GRE DESS sequence (**B**) namely MENSA (Multi-Echo iN Steady-state Acquisition), and a 3D proton-density CUBE sequence (**C**).

Cartilage morphometry refers to a set of imaging techniques that enable quantitative assessment of cartilage morphological characteristics and require high-resolution 3D GRE sequences that yield adequate contrast among the articular cartilage, subchondral bone, menisci, and intra-articular fluid. Over the last two decades, T1-weighted spoiled gradient echo (SPGR, GE) MRI, fast low-angle shot (FLASH, Siemens Healthineers, Erlangen, Germany), fast field echo (FFE, Philips Healthcare, Hamburg, Germany), or water-selective cartilage (WATSc, Philips Healthcare, Hamburg, Germany), associated with fat-suppression techniques to reduce chemical shift artifacts, have been the most commonly used sequences for quantitative morphological assessment due to their high accuracy for the evaluation of 3D cartilage volume, thickness, and area measurements [15]. In more recent times, DESS imaging at 3T has demonstrated the ability to achieve precise and accurate quantitative assessment of cartilage morphology in both individuals with and without knee OA (Figure 3).

To be suitable for quantitative assessment of cartilage status, a magnetic field strength of at least 1.5T is necessary to guarantee a satisfactory signal-to-noise ratio and resolution within a reasonable scanning time. An adequate signal-to-noise ratio (SNR) and contrast-to-noise ratio (CNR) are essential to accurately define the bone–cartilage interface and the articular surface in both healthy and diseased joints, and to avoid significant artifacts such as geometric distortion or signal distortion [16]. The SNR is a crucial attribute of MRI units. It compares the level of a desired signal to the level of background noise, enabling comparison between different machines. An extension of SNR is the CNR, which is a clinically significant indicator of scanner performance. Indeed, the reliability of qualitative and quantitative MRI assessments is partially dependent on the contrast in the images between the tissues of interest. Furthermore, quantitative cartilage imaging requires high-resolution images, with a slice thickness ranging from 0.7 to 1.5 mm and in-plane resolution from 0.3 × 0.3 mm to 0.5 × 0.5 mm, to effectively detect and track small defects measuring less than 5 mm^2^ Moreover, images should be acquired within an acceptable examination time (less than 20 min per pulse sequence) to prevent motion artifacts, ensure patient comfort, and manage expenses [16]. To extract quantitative data from sequential adjacent images, it is necessary to perform segmentation of the articular cartilage first. Over the past two decades, a variety of semi- and fully automated segmentation methods have been suggested for the segmentation of articular cartilage. These methods encompass techniques such as b-spline snakes, edge tracking, local area cartilage segmentation, shape-based methods, clustering approaches, and deep learning-based techniques [17]. Every segmentation approach, whether automated or manual, must undergo rigorous validation to establish the trustworthiness and precision of the outcomes.

Cartilage morphometry can be obtained from individual cartilage plates, as well as from combined regions (such as the medial femorotibial compartment, often affected in OA), or even from specific cartilage subregions. The choice of a particular region of interest for outcome measurement should typically align with the enrollment criteria to prevent regions of interest that are either too expansive or too limited in scope.

In the past, Eckstein et al. and Wang et al. compared numerous data from the literature, confirming the validity (high degree of accuracy), reproducibility (sufficient degree of precision), and sensitivity to OA-related changes of quantitative cartilage volume and thickness assessment. Furthermore, in recent times, quantitative assessments of cartilage morphology have been shown to be sensitive to treatment-related effects by observing a dose–response relationship in femorotibial cartilage thickness changes during the Sprifermin phase 2 trial over 2 and 5 years [18,19]. In the future, following the successful development and approval of a disease-modifying OA drug, automated segmentation techniques relying on machine learning could potentially enable the utilization of quantitative cartilage morphometry for monitoring the treatment progress in individual OA patients [17].

Quantitative morphological analysis of cartilage has also been applied in the field of rheumatology. Specifically, the use of the FLASH sequence to monitor changes in cartilage volume over time in patients with rheumatoid arthritis has shown consistent results [20].

It remains uncertain whether cartilage segmentation is feasible in smaller finger joints. Nevertheless, for longitudinal assessments, the adoption of quantitative morphological MRI techniques is recommended [12].

### 2.3. Semiquantitative (Morphological Changes and Lesion Appearance)

**Figure 4:**
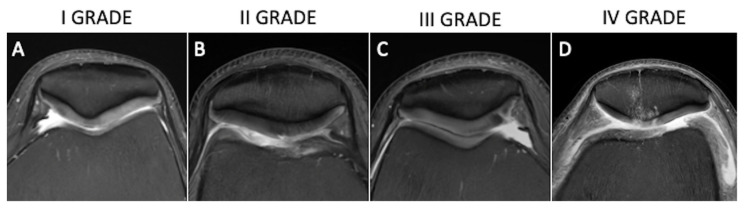
Axial fat-saturated proton-density weighted (**A**–**D**) images depicting hyperintense cartilage with normal contours (**A**, Outerbridge grade 1), defect up to 50% of cartilage thickness (**B**, Outerbridge grade 2), chondral defect >50% (**C**, Outerbridge grade 3), and full-thickness chondral defect with bone uncovering and reactive bone edema (**D**, Outerbridge 4).

Several semiquantitative scoring methods have been developed to obtain homogeneous and standardized assessments of the joint cartilage. In the past, cartilage visualization and evaluation could only be performed arthroscopically, and in the 1960s the Outerbridge classification was introduced [21]. This scoring system was modified with the introduction of MRI and nowadays is part of daily radiological routine. It describes four grades of cartilage damage: grade 1, which involves focal areas of hyperintensity with normal cartilage contours; grade 2 defects, which affect up to 50% of cartilage thickness; grade 3 defects, which affect >50%; grade 4, which occurs at full thickness with bone uncovering and reactive changes in terms of bone edema (Figure 4) [22].

The majority of scoring systems have a focus on OA, but some aim to monitor treatment efficacy in non-OA cartilage defects. To achieve this result, the MOCART (Magnetic Resonance Observation of Cartilage Repair Tissue) score was developed and recently updated with a revised version the MOCART 2.0 [23]. Some scores are joint-specific, in particular knee-specific because this is one of the joints most affected by OA. Unfortunately, this score has some limitations in joints with very thin cartilage (i.e., ankle), in which the intra- and inter-observer reproducibility of this tool has been shown to be highly variable [24,25].

OA can be viewed as an organ failure disease, where the deterioration of one joint leads to a cascading decline in other structures that are biomechanically connected. The WORMS score is a whole organ score, focusing on the structures believed to play a role in the progression of OA. It divides the knee in 14 compartments and scores each on a predefined scale for cartilage, bone, synovitis, and loose bodies [26]. The main drawback of this score is that it is highly time-consuming, requiring a lengthy evaluation time. In 2005, the Knee Osteoarthritis Scoring System (KOSS) score was developed; it is a simple assessment of OA-induced lesion of effusion, cartilage, bone, and meniscal lesions, but it does not assess ligaments [27]. In 2008, the Boston–Leeds Osteoarthritis Knee Score (BLOKS) score was developed with the aim of achieving a better correlation with the Visual Analogue Scale (VAS) [28]. BLOKS demonstrated an inferior efficacy in scoring bone marrow edema-like lesions (BMEL) compared to WORMS, but a superior meniscal scoring [29]. These results led to the development of MRI Osteoarthritis Knee Score (MOAKS) [30] which considers the same 14 subregions as WORMS. Lastly, a scoring method for fast detection of OA phenotypes was developed in 2020: the Rapid OsteoArthritis MRI Eligibility Score (ROAMES), which was adapted from MOAKS and WORMS scores. It is based on a three-compartment approach (patellofemoral joint, medial tibiofemoral joint, and lateral tibiofemoral joint), assessing the maximum grades of cartilage lesions, BMEL, osteophytes, menisci, and inflammation for each joint [31]. All these scores are imaging-based methods that are used to assess OA stage on MRI. Nevertheless, there is not a single best score, as each one has specific characteristics: some are primarily based on imaging, others on clinical correlation. In daily practice, it can be useful to be aware of these different scoring systems to provide the clinician with the most accurate information when it is requested for clinical or research purposes.

### 2.4. Compositional MRI

Articular cartilage is composed of 70–80% water and 20–30% solid extracellular matrix (ECM) with interspersed chondrocytes (approximately 2%) [32]. The ECM consists of collagen and proteoglycans (PG). PG has a core protein bond to one or more glycosaminoglycans (GAGs) [33]. MRI may allow the detection of these biochemical molecules and their changes in pathologic conditions; for instance, in OA. Thus, MRI manufacturers have been constantly developing new MRI techniques to analyze cartilage macromolecules content, with a special focus on collagen and GAGs. Standard MRI sequences are well established [34]; on the other hand, these relatively novel techniques are not routinely used in daily clinical practice. These techniques that allow us to convert MRI relaxation times in quantitative values of tissue provide MRI maps and may reveal minimal changes in cartilage composition even before morphological changes occur [32]. The available MRI mapping techniques are based on different relaxation times and most do not require contrast injection [32,33,35].

### 2.5. T2-Mapping

**Figure 5:**
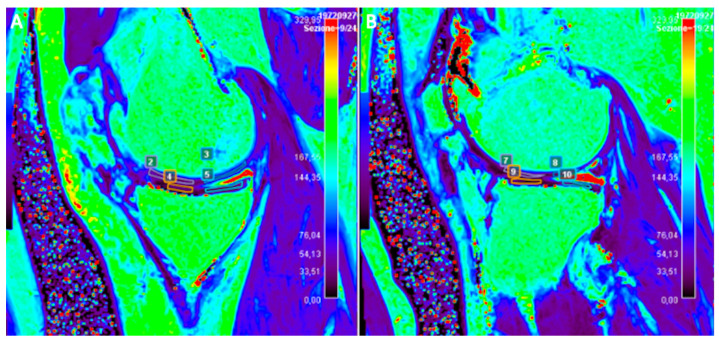
Sagittal T2 map of the right knee of a 47-year-old male patient subjected to anterior cruciate ligament reconstruction. Note the ROIs manually drawn to evaluate T2 relaxation time measurements of joint cartilage on both medial (**A**) and lateral (**B**) femoro-tibial compartments.

Spin-echo T2w sequences allow the visualization of the hydration of the articular cartilage. Normal healthy cartilage has a laminar appearance with a hypointense signal in the superficial and deep zones due to packed collagen fibers and a hyperintense signal in the central transitional zone due to randomly oriented collagen fibers. In degenerated conditions, there is a focal or diffuse increase in T2 signal intensity because collagen fibers acquire a more irregular orientation with an increase in water content in the early stages [32,33]. The PG content instead decreases progressively from early to advanced stages [32,33]. The T2 mapping technique may have several applications in the musculoskeletal field (Figure 5).

This technique has shown high reproducibility (71–88%) in healthy subjects [36,37] and it has been used in previous studies on OA and inflammatory arthritis [38,39]. T2 maps can be calculated using SE sequences with different echo times (ETs). The most used SE sequences are single-echo spin-echo but other 2D sequences have been used, such as FSE, multi-echo spin-echo, and turbo gradient spin-echo. Three-dimensional sequences with isotropic voxels such as DESS have also been used [40]. Another quantitative method is Synthetic MRI, which estimates T2 transverse relaxation using a single saturation recovery TSE sequence [41]. T2* sequences are GRE-based sequences which are susceptible to magnetic field inhomogeneities that, through the acquisition of T2 images at varying ETs, allow us to assess hydration changes with more sensitivity than can be achieved with T2 sequences [42]. T2* mapping uses shorter echo times than T2 mapping, showing a wider range of T2 relaxations in cartilaginous tissue [43]. A recent technique is Ultrashort echo-time enhanced T2* (UTE-T2*) mapping, which has the potential to visualize deep cartilage better than standard T2 mapping. Some studies demonstrated good feasibility of UTE-T2* mapping in detecting cartilage degeneration and a good correlation with morphological cartilage damage [44,45]. These mapping sequences allow us to calculate the decay time between TEs and signal intensity. This calculation is made for each echo time in each voxel. The resulting data are then analyzed by calculating the contrast (measure of the difference of values of neighboring pixels), variance (distribution of pixel respect to the mean), and entropy (measure of the disorder in the distribution of pixels in the image). High contrast, variance, and entropy indicate an altered cartilage structure and were confirmed in vivo in OA subjects compared to normal controls [46]. The laminar analysis of normal cartilage reveals higher T2 values in the superficial zone than in the transitional because the proton mobility is reduced in dense deep zones [47]. In this regard, a study by Mosher et al. [48] demonstrated that senescent changes in the cartilage matrix begin near the articular surface and progressed to deeper cartilage. In addition to age, other factors are involved in cartilage changes over time. A study by Baum et al. [46] has demonstrated that obese subjects have higher mean T2 values, elevated entropy, and more heterogeneous cartilage compared to healthy controls (*p* < 0.05). Further, a study by Friedrich et al. [49] found an association between knee varus malalignment and altered T2 values that lead to unilateral knee OA. The authors reported significantly higher T2 values in the medial compartment (49.44 ± 6.58 ms) than in the lateral compartment (47.15 ± 6.87; *p* = 0.0043) in patients with varus alignment.

Some studies also analyzed the influence of physical activity and lifestyle on cartilage status. A recent study by Chen et al. [50] evaluated the influence of walking, running, and stair activity on the cartilage using T1 rho and T2 mapping on 23 young adults immediately after engaging in these activities for 30 min. This study demonstrated that compression loading in the knee is region-specific and the cartilage superficial layer is more compliant to deformation, which accounts for the nonuniform degeneration observed in the clinic. There is a decrease in the T1 rho and T2 mapping values in the most solicited areas because of the temporary reduction in hydration (the T1 rho of the three regions decreased by 5.667%, 5.031%, and 5.491%; T2 decreased correspondingly by 4.923%, 3.889%, and 6.060%). The superficial layers of the lateral patella cartilage and lateral trochlea cartilage, after stair activity, and posterior part of medial femoral cartilage, after running, experienced the greatest reductions, indicating that these regions may experience greater stress forces and may be more vulnerable to damage in the long term.

### 2.6. T1ρ

**Figure 6:**
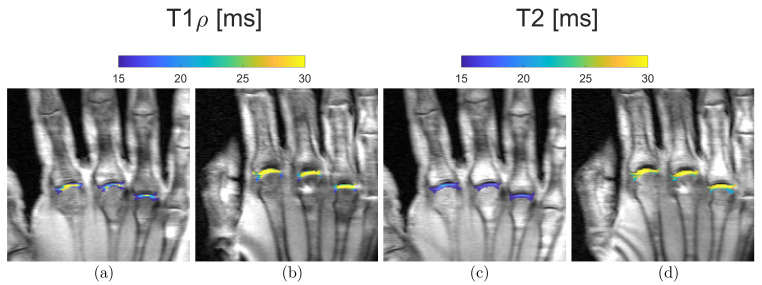
Representative quantitative mapping of relaxation times T1ρ (**a**,**b**) and T2 (**c**,**d**) of a 21-year-old (**a**,**c**) and a 55-year-old subject (**b**,**d**). Blue, i.e., low T1ρ and T2 values, can be seen in the MCP joints of the younger volunteers. In contrast, yellow, i.e., high T1ρ and T2 values, predominate in the MCP joints of the older participant.

T1ρ relaxation time describes spin-lattice relaxation in the rotation frame at the presence of an external radiofrequency (RF) pulse in the transverse plane. An external RF pulse called a spin-lock pulse is applied slowing the magnetization relaxation process in the transverse plane by forcing the spins process along its direction. This process leads to a T1ρ relaxation time longer than the T2 relaxation time. T1ρ imaging allows to quantify and to evaluate the tissue content of low-frequency motional biological components such as proteins. T1ρ is utilized in three different modalities. T1ρ weighted contrast imaging is the basic form and generates qualitative images. T1ρ mapping is the most frequently used, it involves at least two spin-lock times to obtain images with different levels of T1ρ weighted contrast and T1ρ voxel values which are quantitative and independent of the acquisition sequence. T1ρ dispersion is a tissue property that provides a representation of the tissue at low frequencies, reflecting protein content and tissue composition (Figure 6) [51].

T1ρ imaging requires no contrast, no special hardware and allows the usage of different pulse sequences to create a T1ρ weighted magnetization preparation pulse. However, pulse sequence design must be adapted to this quantitative imaging method because the signal evolution during imaging could complicate the quantification. Several pulse sequences have been used for T1ρ imaging. The most frequently used are FSE or TSE [52,53], and even balanced GRE sequences [54]. Technical challenges include the relatively high specific absorption rate (SAR) and the relatively long acquisition time. T1ρ is positively related to the strength of spin-lock field strength, and the higher the MRI field the higher the SAR, which is the amount of RF energy per unit mass per unit time, quantified in W/Kg. To reduce the SAR levels, the acquisition is often significantly lengthened, making this quite a time-consuming technique to use in clinical practice.

Several clinical studies have been conducted on T1ρ imaging. In vitro studies found a strong correlation (r^2^ = 0.987, slope = 0.95) between changes in PG concentration and T1ρ [55], which is a more sensitive and specific tool than T2, resulting in less “magic angle effect” and less laminar appearance [56]. T1ρ values correlate with the biomechanical properties of cartilage (r^2^ = 0.828–0.862) and with clinical and histological grades of degeneration and GAG contents in OA (r^2^ = 0.926) [57,58,59]. In vivo studies demonstrated an excellent reproducibility (average coefficient of variation 4.8%) with a significant increase in T1ρ values with age (r = 0.467, *p* < 0.01) and with OA-related cartilage changes (53.07 ± 4.60 ms) compared to controls (45.04 ± 2.59 ms; *p* = 0.002) [60].

T1ρ imaging has been used in active healthy patients to identify subjects at higher risk of cartilage pathology because it can detect early biochemical changes in the cartilage matrix due to its higher sensitivity than T2 values in detecting PG changes in the matrix. In patients with patellofemoral pain, T1ρ values of the lateral facets of patients with pain and patellar tilt were significantly higher than those of control subjects (46.33 ± 4.92 ms vs. 42.32 ± 3.67 ms, respectively; *p* = 0.031); further, T1ρ values correlated to clinical symptoms and to the degree of patellar tilt (r = 0.72) without relevant T2 values changes [61]. T1ρ values seem to increase in subjects with meniscal tear and in cartilage overlying the BMEL of patients with anterior cruciate ligament tear than in the surrounding cartilage (*p* < 0.001), suggesting this condition may be correlated with and thus predictive of the disease severity of OA [62,63].

T1ρ imaging can also be used to quantitatively assess the cartilage response to loading in vivo. A study by Luke et al. [64] reported significant T1ρ (37.0 to 38.9 ms, *p* < 0.001) values in asymptomatic runners within 48 h after running without morphologic MRI changes. In contrast to the finding of this investigation, a recent study reported a significant decrease in femoral cartilage T1ρ relaxation times immediately after running 3 (65 ± 3 ms vs. 62 ± 3 ms; *p* = 0.04) and 10 (69 ± 4 ms vs. 62 ± 3 ms; *p* < 0.001) miles if compared with baseline T1ρ values. The same study found that changes to the relative PG concentration of knee cartilage due to water flow were mitigated within 24 h. A possible explanation for this is that the prescribed loading magnitude and duration, as well as the time elapsed between loading and the MRI scans, could play a role in modulating T1ρ relaxation times. In patients with Kellgren–Lawrence I-III OA, it has been found that the medial tibial cartilage had higher T1ρ values than the lateral cartilage (medial compartment 59 ± 8 ms vs. 46 ± 7 ms of lateral with *p* = 0.0158) with an association with knee alignment due to different loading to different compartments [65]. The articular surface is rarely homogeneous, and recent studies demonstrated that T1ρ showed more angular dependence than T2 values [66]. T1ρ and T2 values showed different subregional values and angular dependence in asymptomatic knee cartilage with a weak correlation of T1ρ values with T2 measurements (r = 0.217, *p* = 0.127), so awareness of these differences may aid in assessment of cartilage in a specific subregion of the knee [66].

### 2.7. dGEMRIC—Delayed Gadolinium-Enhanced MRI

dGEMRIC is a T1-mapping sequence that can quantitatively assess the GAG content of articular cartilage using the intravascular or intrarticular injection of T1-shortening contrast agent gadolinium diethylene triamine pentaacetic acid (Gd-DTPA2-). Cartilage GAGs are negatively charged molecules, and in normal healthy conditions they repulse the negatively charged molecules of Gd-DTPA2-. When cartilage is damaged, the disruption of the GAGs layer allows the diffusion of Gd-DTPA2- molecules into the cartilage with a consequent shortening of the T1 relaxation time. Therefore, the contrast agent is distributed in inverse proportion to the local PG concentration. The dGEMRIC index measures the concentration of Gd-DTPA2- per voxel and is calculated from five different inversion times using a curve-fitting method. This technique can also be applied to the menisci and is called dGEMRIM, but its use is debated in evaluating meniscal degeneration.

An important point to be considered is the zonal variation of T1 relaxation time as a result of the different content of GAGs [67], which is usually higher in the lateral compartment and can be increased by exercise [68]. The weight-bearing areas of the joint have higher content of GAGs; indeed, a topographic variation in T1, T2 and T2* times has been proven [69]. dGEMRIC mapping can be used in the non-invasive evaluation of cartilage repair following regenerative cartilage treatment. In the follow-up of cartilage lesions of the knee treated by microfracture or autologous chondrocyte implantation, dGEMRIC has been demonstrated as a useful method, achieving local cartilage repair in a focal defect 1 year after treatment (baseline defect 468 ± 91 ms, follow-up 622 ± 241 ms; *p* < 0.01). The same study also demonstrated a local improvement in the dGEMRIC index that was directly related to the improvement of cartilage quality in other joint compartments (*p* < 0.007) [70].

In obese patients, weight loss is associated with an increase in PG content, as demonstrated by a reduction in T1 values in dGEMRIC in the medial knee compartment cartilage (β = 3.9, r2 = 0.26; *p* = 0.008) [71].

The cartilage of some joints is very thin and not so easily distinguished by subchondral bone, making it difficult to perform an accurate evaluation of chondral degenerative changes with mappings, particularly at 1.5T. A recent study [72] assessed the feasibility of the dGEMRIC sequence after the intravascular administration of 0.2 mmol/kg Gd-DTPA2- to study the hip cartilage at 7T MRI, which has a very high spatial resolution owing to increased SNR. This study also demonstrated no significant advantage of using a pre-contrast T1 mapping. There are some drawbacks of dGEMRIC. First, this technique involves administering a dose of 0.2 mmol/kg, which is twice the usual amount, and can contribute to the side effects caused by the gadolinium-induced adverse reactions, nephrotoxicity, or the gadolinium accumulation in the body [73,74]. Then, the scans are typically performed 90 min after intravenous injection to allow the diffusion and equilibrium of the contrast within the cartilage. However, every joint has a proper time delay and is influenced by the anatomy of the different intraarticular components and by the collagen content of the articular cartilage. Lastly, the Gd-DTPA2- distribution is influenced by the different physiological collagen content of the articular cartilage, so T1 relaxation time values after contrast injection must not be related directly to GAGs concentration [70].

### 2.8. Sodium MRI

Sodium MRI (23Na-MRI) is a relatively novel modality that allows non-invasive metabolic imaging. The 23Na ion has a fundamental role in cellular physiology and osmoregulation. Intracellular sodium concentration is much higher than extracellular (in the order of ten times, 10–15 vs. 100–150 mmol/L) and this intra-extracellular concentration gradient is maintained by the Na^+^/K^+^-ATPase pump. This ionic distribution determines the resting membrane potential (60–70 mV) and serves as the basis for the action potentials of neurotransmission in the neurons. In cartilage, 23Na ions contribute to the generation of the osmotic pressure generating the electrostatic repulsion of the sulphate and carboxyl groups of the GAGs, providing compressive elasticity. For quantitative measurements, phantoms with known sodium concentrations may be placed close to the organ under investigation and may provide absolute tissue sodium concentrations. The concentration of sodium within normal cartilage ECM is as high as 300 mM and is in proportion to the PG concentration, which is relatively unaffected by the sodium content or inflammation of the synovium [75].

Sodium imaging remains challenging due to many factors. One of the most relevant is the low SNR of sodium (around 9% of the proton 1H sensitivity) due to its low concentration, short relaxation times, and low gyromagnetic ratio, which make the sodium MR signal almost undetectable. Most of these limitations are abolished when using high-field 3T and ultrahigh-field 7T MRI scanners. Most of the sodium signal is lost in a few milliseconds, thereby resulting in short TR and TE. UTE sequences may overcome the challenge of the very short T2 of 23Na ions at the cost of image blurring and a decrease in SNR. An important limitation is that the difference between intra- and extracellular sodium signals cannot be established by relying only on relaxation time constant characteristics; further, the cartilage is usually very thin and immersed in synovial fluid (which has its own sodium concentration); therefore, it is necessary to avoid the partial volume effects to achieve a correct estimation of the sodium parameters of cartilage [76]. Sodium imaging also requires specialized RF coils and customized UTE sequences to detect signals, and many are still in development [77].

Sodium MRI is a promising technique for imaging the integrity of articular cartilage in vivo. Several studies performed in vitro and on animals have proved that non-invasive sodium MRI can directly determine the cartilage GAG content and their depletion in OA with a subsequent reduction in cartilage sodium content. In OA, sodium content is thought to decrease progressively, but a recent study [78] found higher 23Na values in OA patients at all timepoints (baseline and at 3 and 6 months) in an age-matched cohort comparison with healthy control subjects using T1-weighted sodium MRI. A UTE T1 short TR sequence would preferentially attenuate the fluid signal over the cartilage signal, removing the confounding synovial fluid signal. Data were compared to clinical (VAS, KOOS) and imaging (Kellgren–Lawrence) scores and revealed a weak relationship (r = 0.31) with sodium concentration over the entire knee [78]. Moreover, sodium MRI was found to be a poor method of assessing articular cartilage stiffness [76], but the results are still subject to debate.

Sodium imaging has also been applied to the evaluation of cartilage and repair tissue in patients after various cartilage repair surgery techniques [79]. One of the first studies on the evaluation of cartilage repair showed that sodium MRI could allow the differentiation between matrix-associated autologous chondrocyte transplantation (MACT) repair tissue and native cartilage of patients without the need for contrast agent application. However, in the early post-operative period, the repaired cartilage may have higher water content and the results could be slightly altered [79]; therefore, it would be advisable to use fluid-suppressed sodium MRI sequences.

Hence, sodium MRI remains a promising technique for non-invasive evaluation of articular cartilage and repair tissue, but further research and technical developments are warranted to improve its accuracy and applicability in clinical practice.

### 2.9. Diffusion-Weighted Imaging

Diffusion-weighted imaging of the cartilage is based on diffusion tensor imaging (DTI), which is sensitive to the movement of water molecules in different directions and has been used in several different applications as well as in the musculoskeletal system [80]. DTI can provide information about the two main components of the cartilage matrix: collagen and PG. An important tool in DTI is the apparent diffusion coefficient (ADC) which is a measurement of dispersion of water molecules in a specific diffusion time due to interaction with macromolecular matrix [77]. Cartilage macromolecules restrict the diffusion of water and lower the ADC values, which are therefore lower than in bulk water [81]. DTI measures two parameters: mean diffusivity and fractional anisotropy. Mean diffusivity reflects the average diffusion of water molecules in all directions and is influenced by the PG concentration. Fractional anisotropy reflects the degree of directionality of water diffusion and is influenced by the collagen structure. DTI can detect changes in these parameters, which reflect PG and collagen depletion, which are associated with cartilage degeneration or OA [81]. Ex vivo [82] and in vivo [83] studies demonstrated that DTI is highly sensitive for the detection of early cartilage degeneration, with accuracy of 95% ex vivo [82], sensitivity of 86%, and specificity of 89% in vivo [83]. In OA, the significantly decreased fractional anisotropy values suggest that alterations in the structure of collagen may take place at an early stage of cartilage deterioration [82]. To perform DTI of the cartilage, some technical challenges must be overcome. Indeed, cartilage is a very thin structure with a low T2 relaxation time and DTI has limited SNR, is sensitive to motion, and requires long scan times [77]. The most-used sequences are pulse sequences with steady precession [84], but also double echo SSFP sequences have been tested [85]. Recently, a special pulse sequence called radial imaging spin-echo diffusion (RAISED) has been developed, which allows high-resolution imaging of the cartilage with minimal distortion and motion artifacts using a radial acquisition with 2D echo-planar readout (EPI), which is an echo-planar sequence with high temporal resolution [86]. The RAISED sequence requires a 3T MRI scanner and takes about 15 min to acquire. DTI has proven to be a reliable technique with many applications in the musculoskeletal field with an excellent inter-observer and inter-vendor reliability with ICC around 0.90 (95% CI) [87]. It is a promising and potentially powerful technique that can provide insights into the mechanical integrity and health of the articular cartilage, but DTI needs high-field MRI (3T) and quite long acquisition times, and more studies are needed to validate its accuracy, reproducibility, and clinical relevance.

### 2.10. UTE Ultrashort-Time Echo MRI

**Figure 7:**
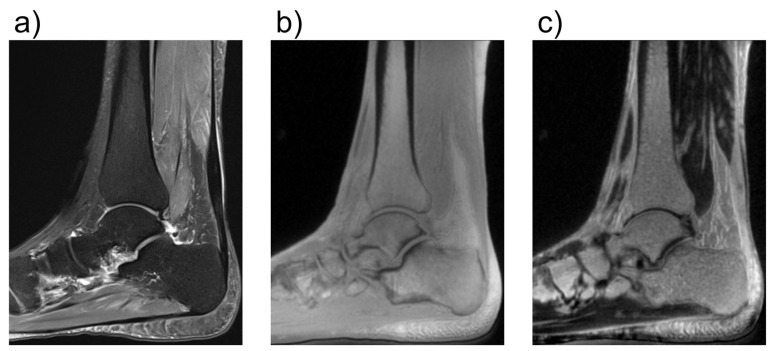
Comparison of conventional and ultrashort echo time (UTE) MRI images of the foot in the sagittal plane. In (**a**), a fat-saturated proton-density weighted image is shown with an echo time (TE) of 42 ms. In (**b**), a UTE image with a TE of 0.05 ms is shown. In (**c**), a subtraction image is shown, in which the signal of an image with TE = 4 ms is subtracted from the image in (**b**). While in (**b**), all structures have very high signal because of the short TE, in (**c**) only the images with very short T2*-times are shown as bright. With regard to cartilage, this enables better assessment of the health of the deepest calcified cartilage layers. The UTE images were acquired using a density-adapted 3D radial (DA-3D-RAD) imaging sequence.

UTE MRI is a powerful imaging technique that allows for the direct visualization of semi-solid tissues with highly organized collagen fibers which usually have short or ultrashort T2 relaxation times [88]. Conventional MRI techniques have been developed to image and quantify tissues and fluids with long transverse relaxation times between 1 and 2 ms, such as muscle, cartilage, liver, white matter, gray matter, spinal cord, and cerebrospinal fluid [77,88]. However, the body also contains many tissues and tissue components such as the osteochondral junction, menisci, ligaments, tendons, bone, lung parenchyma, and myelin, which have short or ultrashort T2s, resulting in a signal decay faster than MRI acquisition. After RF excitation, the transverse magnetizations of these tissues typically decay to zero or near zero before the receiving mode is enabled for spatial encoding with conventional MRI [88]. As a result, these tissues appear dark, and their MR properties are not discernible. However, when UTE is used, signals can be detected from these tissues before they decay to zero. In cartilage imaging, UTE sequences with a TE less than 100 µs are capable of detecting signals from both fast- and slow-relaxing water protons in cartilage, allowing for comprehensive evaluation of all the cartilage layers, especially for the short-T2 layers, which include the deep layers, and in calcified articular zones (Figure 7) [89].

A series of UTE MRI techniques have been developed for high-resolution morphological and quantitative imaging of these short-T2 tissues. These techniques include T1, T2, T2*, T1ρ, magnetization transfer (MT), DESS, quantitative susceptibility mapping, and inversion recovery [90]. Researchers developed imaging sequences that can acquire images with ultrashort echo-times as low as 40 µs. The most-used sequences are GRE sequences with half-excitation RF pulses [91], but recently, a hybrid cartesian and radial imaging sequence called PETRA has been introduced [92], which can detect both long and short TE structures together. Another advantage of using UTE imaging with ultrashort echo times is increased robustness towards susceptibility artifacts. Those artifacts can arise on borders between tissue and or when there is very fast motion affecting image interpretation. UTE imaging, in particular UTE Adiabatic T1ρ and UTE-MT modeling techniques, has shown good performance and lower sensitivity to the magic angle effect than conventional quantitative MRI techniques. UTE MRI has also been demonstrated to be reliable in assessments of cartilage endplate damage and lumbar intervertebral disc degeneration in patients with chronic low back pain with excellent inter-observer agreement (k = 0.839, *p* < 0.001) [93]. Hence, UTE MRI is a valuable tool for assessing tissues with short or ultrashort T2 relaxation times such as menisci and the deep layers of articular cartilage. Its clinical value still needs to be proven by in vivo studies.

### 2.11. GAG-CEST—Glycosaminoglycan Chemical Exchange Saturation Transfer Imaging

In a typical MRI sequence, the signal we observe primarily stems from the hydrogen nuclei within unrestricted water molecules.

**Figure 8:**
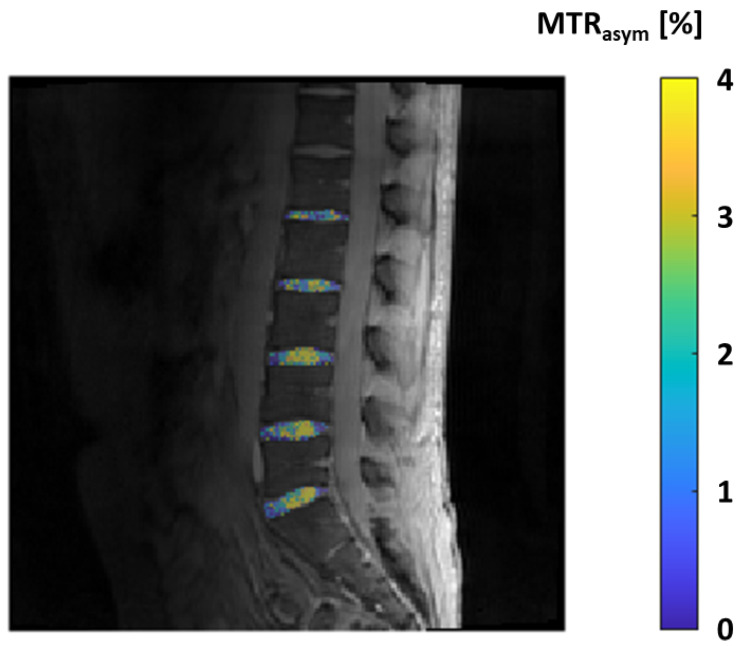
GAG-CEST effect in the lumbar intervertebral discs expressed by the parameter MTRasym in percent, measured in a 25-year-old healthy male volunteer.

However, in an MT sequence, a preliminary saturation pulse is administered before the primary MRI sequence. This pulse specifically energizes the broad signal emanating from water molecules bound to less mobile macromolecules. The interaction between these two pools of water causes a reduction in the signal strength of the unrestricted water, with the degree of attenuation contingent on the dynamics of the exchange process and the size of the bound water pool. MT is frequently quantified using the MT ratio, which is straightforwardly calculated by comparing the signal intensities detected with and without the preparatory saturation pulse applied. In the context of cartilage imaging, the critical MT interaction primarily occurs between the bulk water and the water molecules bound to the collagen fibers within the cartilage extracellular matrix. A modern advancement based on the MT principle is the technique known as chemical exchange-dependent saturation transfer (CEST). This technique involves the selective excitation of exchangeable protons within a solute, and as these protons chemically exchange with the protons in water, it leads to a noticeable reduction in the magnetization of the overall water pool [94]. Within articular cartilage, a selective excitation of the hydroxyl residues on GAGs is employed to create contrast between areas with varying GAG content, a technique known as GAG-CEST [95]. This approach allows for the direct quantification of GAG content, typically expressed as an MT asymmetry value or percentage. Regions characterized by lower GAG content exhibit reduced MT and consequently lower asymmetry values [96]. Soellner et al. have shown that GAG-CEST imaging effectively mirrors the GAG content and holds promise as a diagnostic tool for identifying initial knee-joint cartilage damage and distinguishing between different International Cartilage Repair Society grades through non-invasive MRI, even in the early stages of clinical application (Figure 8) [97].

This technique has been successfully applied in a clinical environment at 3T and demonstrated comparable results to dGEMRIC and T2 mapping in the detection of both normal and damaged cartilage with non-significant differences at the comparison of the areas under the curve using ROC analysis (*p* = 0.14 for T2 mapping vs. CEST, *p* = 0.89 for CEST vs. dGEMRIC) [98]. A fast 3D GAG-CEST sequence applied at 7T, acquired within a clinically feasible scan time of 7 min, has been demonstrated to be clinically applicable and capable of differentiating healthy from damaged cartilage (*p* < 0.05) in patients before their cartilage repair surgery with good to excellent reproducibility (ICC = 0.87–0.97) [99]. In conclusion, GagCEST holds significant potential to expand its role in both research and clinical settings in the future. All quantitative MRI techniques are described in Table 1.

## 3. Implementation and Significance for Clinical Practice

With the constantly improving technical options in MRI, the joint cartilage quality can now be examined validly and reliably using numerous sequences as described. Initially, these were very technical studies which showed that these new sequences truly reflect the local histology. This was followed by numerous clinical studies that were also able to investigate the specific outcome of degenerative and inflammatory joint diseases. A transition to the clinical context was thus achieved.

All these points are of high clinical relevance. Today, it is clear that even preliminary stages or very early signs of degenerative chondral disease must be recognized immediately so that all treatment options can be considered and discussed with the patient. The same applies to inflammatory rheumatic joint diseases. It has been shown that changes in the cartilage are very early signs of impending osseous damage. The extent of local inflammation (synovial inflammation) clearly correlates with the loss of cartilage quality. To a certain extent, all of this happens before osseous affections become apparent in conventional MRI, e.g., as bone marrow edema. In addition, such cartilage changes are predictive of a poorer therapeutic outcome. All these advances in research, which nowadays make it increasingly feasible to examine cartilage non-invasively using MRI without using contrast agents, are progressively leading to the techniques being used not only in clinical studies but also very specifically in a clinical context as a supplementary sequence in a standardized MRI of a joint. Even if this is currently only happening in large centers, the potential acquisition of information is extremely important, allowing healthcare professionals to provide patients with the best possible advice. It is a further step towards recognizing very early changes that occur long before the conventional X-ray image used as the gold standard in the past and actively integrating these into the decision-making process, which will allow patients’ symptoms to be recognized as early as possible and then will allow healthcare professionals to closely monitor those at high risk. On the other hand, however, it will certainly also enable us to better understand the local processes and, if necessary, to create individualized treatment concepts as part of personalized medicine. MRI techniques of the articular cartilage will play an even more important role in the future, but some limitations must be addressed before these advanced tools can be employed in clinical practice. Most of all, standardization of technical parameters of imaging protocols is essential to make these sequences reproducible and to allow them to be objectively assessed in different centers. Further, normative values based on age, gender, and sport activities are required, along with well-established cut-off values specifically proven for each joint, to make quantitative data robust and applicable. To achieve this, large multicenter prospective studies are warranted to avoid the risk that the influx of technology will not find an outlet in daily routines.

## 4. Conclusions

We have reviewed the current updates in compositional and quantitative cartilage MRI provided by the most recent sequences and techniques. The discussion encompassed both the possible hurdles and prospects associated with emerging techniques, addressing issues such as standardization across various scanners, vendors, and institutions. The actual challenge will translating several of these advancing cartilage MRI techniques into broader clinical application in the near future. This translation is poised to enhance the detection of disease onset and progression, improve treatment monitoring, and ultimately contribute to more effective patient care management.

## Figures and Tables

**Table 1:** Pros and cons of quantitative MRI sequences and techniques.

Techinque	Sequences Used/Component Assessed	Pros	Cons
**T2-mapping**	T2*, UTE-T2*, T2w SE, FSE, Multi Echo SE, Turbo Gradient SE, DESS Cartilage hydratation, Cartilage, Water	High reproducibility. T2* and UTE sequences allow a better visualization of deep cartilage and osteochondral junction. Predictive for OA in areas with much compression loading. No contrast administration.	Susceptible of magic angle artifact and magnetic field inhomogeneity.
**T1ρ**	FSE, TSE, balanced GRE Tissue proteins and composition, GAGs	Quantitative and sequence-independent voxel values. Excellent reproducibility and sensitivity in detecting GAGs depletion. No contrast administration. No special hardware needed.	High SAR, long acquisition time, pulse sequence must be adapted.
**dGEMRIC (delayed gadolinium-enhanced)**	T1-mapping after gadolinium administration GAG content	Evaluation of cartilage repair following regenerative treatments. Suitable for visualization of osteo-chondral interface and zonal variation of GAG content.	Intravascular or intrarticular administration of contrast. No standard time for intracartilage diffusion (usually after 90 min). Values not directly correlated with GAG concentration.
**Sodium MRI**	UTE T1w Na+ concentration of cartilage ECM	Promising technique for non-invasive evaluation of articular cartilage and repair tissue. Direct correlation with Na+ cartilage content. No contrast administration.	Low Na SNR, need of 3T or higher. Partial volume effect for intra- and extra-cellular sodium signals. Need of specialized RF coils and customized UTE sequences.
**DTI (Diffusion Tensor Imaging)**	Pulse sequences with steady precession, double echo SSFO, RAISED Collagen, PG	High sensitivity and specificity detecting early cartilage degeneration and collagen structure alteration. No contrast administration.	Limited SNR, motion sensitive, long acquisition time. High-field MRI (3T).
**UTE (Ultrashort time echo) MRI**	T1, T2, T2*, T1ρ, magnetization transfer (MT), DESS, IR, quantitative susceptibility mapping Cartilage deep layers, osteochondral junction	Assessment of thin articular structures with short-T2 other than long-T2 structure. Lower susceptibility artifacts and magic angle effect than conventional quantitative MRI. Excellent interobserver agreement to cartilage endplate damage and intervertebral disc degeneration.	Not well validated. Inhomogeneous data on compositional quantification.
**GAG-CEST (glycosaminoglycan chemical exchange saturation transfer imaging)**	Magnetization transfer (MT)	Direct quantification of GAG content. Promising for identification of initial knee-joint cartilage damage. Comparable to dGEMRIC and T2 mapping. Good to excellent reproducibility at 7T MRI.	High-field MRI (3T or more). Not yet well validated.

## Data Availability

No new data were created or analyzed in this study. Data sharing is not applicable to this article.
